# Unexpected new insights into DNA clamp loaders: Eukaryotic clamp loaders contain a second DNA site for recessed 5′ ends that facilitates repair and signals DNA damage

**DOI:** 10.1002/bies.202200154

**Published:** 2022-09-18

**Authors:** Huilin Li, Mike O’Donnell, Brian Kelch

**Affiliations:** 1 Department of Structural Biology, Van Andel Institute, Grand Rapids, Michigan, USA; 2 DNA Replication Laboratory, The Rockefeller University, New York, New York, USA; 3 Howard Hughes Medical Institute, The Rockefeller University, New York, New York, USA; 4 Department of Biochemistry and Molecular Biotechnology, University of Massachusetts Chan Medical School, Worcester, Massachusetts, USA

**Keywords:** AAA+ oligomers, 9–1–1 clamp, clamp loader, DNA repair, DNA replication, PCNA clamp, sliding clamp

## Abstract

Clamp loaders are pentameric AAA+ assemblies that use ATP to open and close circular DNA sliding clamps around DNA. Clamp loaders show homology in all organisms, from bacteria to human. The eukaryotic PCNA clamp is loaded onto 3′ primed DNA by the replication factor C (RFC) hetero-pentameric clamp loader. Eukaryotes also have three alternative RFC-like clamp loaders (RLCs) in which the Rfc1 subunit is substituted by another protein. One of these is the yeast Rad24-RFC (Rad17-RFC in human) that loads a 9–1–1 heterotrimer clamp onto a recessed 5′ end of DNA. Recent structural studies of Rad24-RFC have discovered an unexpected 5′ DNA binding site on the outside of the clamp loader and reveal how a 5′ end can be utilized for loading the 9–1–1 clamp onto DNA. In light of these results, new studies reveal that RFC also contains a 5′ DNA binding site, which functions in gap repair. These studies also reveal many new features of clamp loaders. As reviewed herein, these recent studies together have transformed our view of the clamp loader mechanism.

## INTRODUCTION

The first protein discovered to encircle DNA was the *Escherichia coli β*-clamp, thus identifying a new class of protein referred to as a DNA polymerase sliding clamp.^[1,2]^ The *β* clamp cannot get onto DNA by itself; for this it requires a pentameric clamp loader that uses ATP hydrolysis to open and close the clamp around DNA. Once on DNA, the *β* clamp binds the DNA polymerase and holds it to DNA, sliding along behind it for exceedingly high processivity during replication.^[2]^ The structure of the *β*-clamp revealed an expected C2 symmetry but unexpected pseudo six-fold symmetry in the dimer because each monomer is composed of three nearly identical domains (Figure 1A).[1] With development of the SV40 in vitro replication system, two protein factors, PCNA and replication factor C (RFC), were observed in eukaryotes that might function as a clamp and clamp loader (Table 1).^[3,4]^ Because eukaryotic PCNA is approximately two thirds the length of the *β*-clamp, PCNA was intuited to be comprised of three two-domain subunits to provide a trimer of C3 symmetry that also contains six-domains that produce a pseudo six-fold symmetric ring like the E. coli *β*-clamp, with RFC being the loader.[1] Indeed, the structures of eukaryotic and archaeal PCNA clamps confirmed this prediction (Figure 1A).^[5–7]^

The similarity of bacterial and eukaryotic sliding clamps extends to their respective clamp loaders. All organisms utilize a clamp loader that requires five subunits for activity; the subunits are homologous to one another and from bacteria to human.^[8,9]^ Each subunit contains the AAA+ fold (ATPases associated with diverse cellular activities) that has two subdomains, the Lid domain and the ATP binding fold.^[10]^ The five subunits are arranged in a spiral, C-shape to form a DNA binding “central chamber”. The five subunits are referred to as A through E, with the A subunit at the base of the spiral. There is a lateral gap between the A and E subunits for DNA entry into the central chamber, and the clamp is bound just below the DNA binding central chamber.^[11,12]^ The binding of ATP drives the clamp opening reaction such that the gap in the clamp is aligned with the gap in the clamp loader, thus positioning the DNA through the lumen of the open clamp. This is illustrated for the canonical clamp loader of eukaryotes in Figure 1B. In typical fashion for AAA+ oligomers, the ATP sites of the clamp loaders are at subunit interfaces.^[13]^ ATP hydrolysis results in the clamp loader ejecting from the clamp–DNA complex, leaving the clamp to function with DNA polymerase (or other factors). These steps are general among all three domains of life^[7]^ and are summarized in the illustration of Figure 1B.

The canonical clamp loader of eukaryotes for DNA replication is the RFC pentamer. Unlike bacteria and archaea, eukaryotes contain three other “RFC-like clamp loaders” or RLCs (RFC-like complexes).^[14]^ These RLCs are: Rad24-RFC (Rad17-RFC in human), Ctf18-RFC, and Egl1-RFC (Table 1). One of these RLCs – Rad24-RFC – assembles a different trimeric ring, the 9–1–1 heterotrimer clamp (yeast Ddc-1-Mec3-Rad17; human Rad9-Hus1-Rad1) onto DNA which functions in the S-phase DNA damage response.^[15–17]^ The function of the other two alternative clamp loaders is not yet fully defined, although they are suggested to be PCNA unloaders.^[18,19]^ However, aPCNAloading function is recently suggested for Ctf18-RFC.^[20,21]^ This review will focus on important new findings of the canonical RFC and a surprising new finding about Rad24-RFC, as illustrated in Figure 1C.

## THE CANONICAL RFC EUKARYOTIC CLAMP LOADER

The canonical eukaryotic clamp loader of PCNA is the RFC hetero-pentamer. Four of the eukaryotic clamp loader subunits, subunits (Rfc2–5) are present in all the alternative clamp loaders. The unique “A subunit” is larger than the other subunits and distinguishes each of the different clamp loaders from each other (Table 1). The Rfc2–5 subunits are sometimes referred to as the small subunits because the A subunits are much larger and contain extra N-terminal and/or C-terminal extensions compared to the small subunits. We illustrate this and the extra domains of Rfc1 and Rad24 in Figure 2. Importantly, the A subunit (Rfc1 and Rad24) contains a C-terminal domain, referred to as A′, that will be explained further below. Each of the four Rfc2–5 “small subunits” has three domains that are homologous to those present in the A subunit, including the “motor” domain of the ATP-dependent clamp loading reaction. The two N-terminal subdomains of Rfc 2–5 comprise the AAA+ motor domain, consisting of the ATP binding and AAA+ Lid subdomains that interact with ATP, and the C-terminal domain that forms a pentameric “collar” that tightly holds the five clamp-loading subunits together. There is a wide gap in the side of the clamp loader of *E. coli* for passage of DNA,^[12]^ but the unique Rfc1 C-terminal A′ domain mostly fills this gap,^[22]^ and this narrowed gap is referred to as the A-gate (composed of the A′ domain and the AAA+ domains of the A subunit).^[23]^ This region of Rfc1 is also mimicked in the T4 clamp loader, although their primary sequences and 3-dimensional structures are quite different.^[23,24]^

The first structures of RFC–PCNA–ATP*γ*S in both yeast and human systems showed that only two PCNA protomers are contacted by RFC, and the PCNA ring remains closed.^[22,25]^ This closed RFC structure obstructs the central chamber, thereby preventing DNA binding. Additionally, a structure of the T4 phage clamp loader with its trimeric gp45 clamp and DNA indicated an opening in the clamp that was not wide enough for dsDNA to pass.^[26]^ The absence of a structure with a wide enough opening for dsDNA passage implicated a “screw-cap” process, in which the clamp is first placed onto ssDNA and then “screwed” down around dsDNA before the ATPase clamp loader is ejected.^[23]^

New cryoEM results have provided information that changes the “screw cap” model to a “direct DNA insertion” model. CryoEM studies of yeast RFC–PCNA–ATP*γ*S identify a new structure in which RFC undergoes a large conformational change and contacts all three PCNA protomers, opening them about 20 Å in a right handed lock-washer spiral, sufficiently wide to accommodate dsDNA.^[24]^ The large conformation change of RFC is sometimes referred to as a “crab claw” motion because the expansion of the pentamer, while it latches onto the three protomers of PCNA, results in ring opening – resembling the opening of a crab claw.^[24,26]^

ATP binding enables the clamp loader to bind and open the sliding clamp. That ATP*γ*S allows RFC to undergo a large conformation change is consistent with earlier predictions based on biochemical and structural studies of clamp loader action.^[11,12,27–30]^ However, ATP binding is not sufficient to mediate the conformational change of RFC to the open form; instead, opening of RFC is conformationally coupled to the opening of PCNA.^[24]^ Thus, both the sliding clamp and clamp loader undergo a concerted crab-claw opening motion. This conformational coupling is proposed to regulate the binding preference of RFC: because RFC only becomes competent to bind DNA in the central chamber when PCNA is opened, RFC will prefer to bind PCNA before binding to primer-template DNA. This binding preference is important physiologically because if RFC bound to DNA, then PCNA binding would be blocked, resulting in a non-functional complex.

Upon DNA binding to RFC–PCNA_open_–ATP*γ*S, the gap in the PCNA ring narrows to approximately 14 Å, as observed by three different labs.^[24,31,32]^ Indeed, the open PCNA appears in equilibrium with a closed PCNA form. Interestingly, the ATP sites appear in a spiral conformation and are competent for hydrolysis in these recent RFC–PCNA–DNA–ATP*γ*S structures. The constriction of the PCNA opening upon binding DNA appears to tighten the interfacial ATP sites, and these slight movements may result in active ATPase catalysis for clamp loading activity.^[24,32]^ The earlier structure of RFC bound to only two protomers of closed PCNA likely captures an early encounter of RFC–PCNA, before clamp opening, in which the tight AAA+ spiral prevents premature ATPase activity.^[22,26]^

Interestingly, superposition of the RFC–PCNA_open_–ATP*γ*S–DNA and RFC–PCNA_closed_–ATP*γ*S-DNA structures show no significant change in the RFC conformation despite the large change in PCNA ring opening (Figure 3). The lack of a substantive conformation change of RFC in these structures suggests that the PCNA ring is in equilibrium between open and closed states while RFC stays relatively static. Thus, it appears that RFC–ATP*γ*S does not require ATP hydrolysis to open/close the PCNA ring. However, ATP hydrolysis is needed for RFC to eject, leaving behind a closed PCNA–DNA complex.^[33–35]^ This scenario is consistent with the biochemical finding that ATP hydrolysis precedes clamp closure.^[36–38]^

Furthermore, high resolution structures of the RFC–DNA interaction show that RFC unexpectedly melts at least 1 bp at the 3′ end of the primed substrate.^[24,31,32]^ This melting activity is specific to RFC, as the bacterial and T4 phage clamp loaders do not melt any DNA in solution or in crystal structures.^[24,26,39]^ The A subunit of RFC contains a “separation pin” at the top of the central chamber that flips the 3′ nucleotide onto aromatic residues that line an exit channel that only appears upon opening of RFC.^[24]^ Unlike most DNA melting proteins, RFC does not require any ATP hydrolysis to melt DNA, which is entirely driven by binding energy. The function of this melting is not fully understood, but likely facilitates PCNA loading at DNA that is nicked or contains a small single-stranded gap (see below). This melting could also be used to confirm recognition of a 3′ terminus.

It is noteworthy that the Rfc1 subunit dominates the DNA binding interface, compared to Rfc subunits 2–5.^[24,31,32]^ Specifically, the Rfc1–DNA interaction surface area accounts for 64% of the buried surface of DNA, compared to 36% of the buried surface area of DNA for the Rfc 2–4 subunits combined.^[24]^ This has important implications to the function of alternative clamp loaders, each of which exchanges a new subunit for Rfc1 (Table 1). Hence, the DNA structure, or the mode of binding and its outcome, could be easily modified by the large subunit of each of the alternative clamp loaders as proposed.^[24,31,32]^ PCNA interaction with DNA may also play a role in the clamp loading reaction.^[40,41]^

## THE EUKARYOTIC Rad24-RFC LOADS THE 9–1–1 CLAMP AT 5′ DNA ENDS

As described earlier in this review, eukaryotes have four different types of clamp loaders, the canonical RFC and three RLCs. The RLCs contain the Rfc 2–5 subunits, and only differ in the Rfc1 subunit (Table 1). For the discussion to follow, we present new and unexpected developments in our knowledge about function of the Rad24-RFC, which is called Rad17-RFC in humans.

Rad24-RFC assists in repairing double-strand breaks (DSBs) by loading the 9–1–1 clamp at 5′ DNA recessed ends that are produced at a DSB or during stalled replisome polymerases that get stuck at lesions and do not complete fill-in at repair gaps, Okazaki fragments or the leading strand, thereby leaving a recessed 5′ end that normally would be only a transient occurrence.^[42,43]^ An early step in responding to DNA damage is the 9–1–1 clamp, which is specifically loaded onto 5′ DNA ends to activate the ATR kinase (yeast Mec1–Ddc2, human ATR–ATRIP) that controls the DNA damage cell cycle checkpoint.^[44]^ 5′ ends are normally ligated and are present only transiently, but if template lesions prevent the completion of an Okazaki fragment, or stop polymerase and prevent continuing the leading strand, the expected subsequent leading strand repriming event (e.g., via PrimPol?) will leave a recessed 5′ end that may act to load 9–1–1 and signal DNA damage. The recent cryoEM structure of the yeast Rad24-RFC–DNA–9–1–1 clamp showed a totally unexpected process by which the Rad24-RFC–9–1–1 DNA clamp/clamp loader engages 5′ recessed DNA.^[45,46]^

Before the Rad24-RFC–9–1–1 clamp–DNA structure was solved, it was generally assumed that the recessed 5′-end DNA binds into the central chamber of the Rad24-RFC clamp loader, similar to recessed 3′-end containing DNA that binds inside the canonical Rfc1-RFC for PCNA loading. However, this long held expectation was not at all the case. Instead, the Rad24-RFC–9–1–1 clamp–DNA structure showed that 5′ DNA binds to an external site on Rad24, rather than inside central chamber of the clamp loader.^[45,46]^ 5′ DNA binding to the external site occurs on Rad24 at a site formed between the collar and AAA+ domains of Rad24 which are connected by a “long linker” (see “LL” in Figure 2).^[46]^ The 3′ ssDNA that emanates from the recessed 5′ end threads into the central chamber of the clamp loader to bind the motor subunits.^[45,46]^ Interestingly, it appears that the central chamber of Rad24-RFC has loop(s) that protrude into the central chamber that perhaps prevent dsDNA from entering inside this RLC, helping to select for a long ssDNA gap.^[45,46]^

These unexpected findings are quite exciting because: (1) it showed the presence of a second DNA binding site in a clamp loader, and (2) it indicated that ssDNA – not just dsDNA – can traverse the central chamber of the clamp loader and trigger ATP hydrolysis of the motor subunits for 9–1–1 clamp loading. Prior to these findings, clamp loaders were thought to require dsDNA inside the central chamber to scaffold and configure the clamp loader subunits for ATP hydrolysis. As described below, the 5′ DNA external site was found to generalize to canonical RFC, and likely generalizes to all alternative RFCs.^[46]^

The Rad24-RFC–911–DNA–ATP*γ*S complex showed a nearly shallow helical configuration of ATP sites that is similar to the RFC–PCNA–DNA–ATP*γ*S complex, and two conformers in which the 9–1–1 ring was either open or closed. Like the RFC–PCNA studies, the Rad24-RFC conformation was essentially unchanged in the open and closed forms of the 9–1–1 clamp. The 9–1–1 clamp can open in-plane as wide as 27 Å, more than sufficient for dsDNA passage.

Thus, both RFC and Rad24-RFC can widely open their respective clamps, but they do so in different ways. While RFC opens PCNA into a shallow lock washer with sufficient distance for dsDNA to enter, the Rad24-RFC keeps the 9–1–1 clamp opening in-plane with a sufficient gap in the clamp for dsDNA to enter (see Figures 3 and 4). Despite the differences between Rad24-RFC–9–1–1–DNA and the RFC–PCNA–DNA structures, their comparison shows they are more alike than distinct, and reinforce new mechanistic insights of how RFC and RFC-like complexes function with clamps.

## RFC BINDS DNA WITH NICKS OR SMALL GAPS USING TWO DNA SITES TO BIND AND MELT THE DUPLEX

Opening of RFC causes the Lid of the A-subunit to stretch out into an “alternative linker” (AL) between the Lid and the collar domains,^[24,32]^ similar to the “long linker (LL)” in the 5′ DNA site observed in the Rad24-RFC structures (see Figure 2).^[45,46]^ This conformational change creates a DNA binding site for a recessed 5′ end in the canonical RFC.^[24,31,32]^ This 5′ DNA site also utilizes the N-terminal BRCT domain of Rfc1 that is outside the AAA+ module and has been demonstrated to preferentially bind DNA with a 5′-phosphate.^[47,48]^

The 5′ DNA site of Rfc1 is not required for cell viability but mutations or deletions of the BRCT result in defective gap repair.^[49–51]^ The possibility that RFC binds a DNA gap, via the BRCT region’s ability to bind a recessed 5′ end, along with the 3′ primer terminus in the central chamber of the entire clamp loader, became obvious from the external DNA binding site in the Rad24-RFC–9–1–1 structures.^[45,46]^ Recent structures from three different labs have shown that this is indeed the case as illustrated in Figure 5.^[31,32,50]^ Consistent with a role in gap repair, the recent cryoEM data also reveal that RFC can even unwind dsDNA at both the internal and external DNA binding sites.^[31,50]^ There appear to be two separation pins(for 3′ [Trp638 and Phe582] and 5′ [His556 and His659] ends) that may coordinate with each other at a nick to unwind up to five nucleotides of duplex DNA for clamp loading.^[24]^

RFC mediated DNA unwinding at a nick is not driven by ATP hydrolysis but through DNA binding energy, and PCNA loading is within a few fold of the same efficiency as being loaded at a nick or gap compared to a 3′ primer template junction.^[32,50]^ Moreover, the intrinsic unwinding activity of RFC allows it to load PCNA at a variety of DNA structures. It seems possible that similar unwinding activities may facilitate other RLCs to function on particular DNA structures that are specific to them. For example, the Elg1-RFC is proposed to be a PCNA clamp unloader that removes PCNA from non-nicked duplex DNA^[19]^; this action could possibly be facilitated by an intrinsic unwinding activity. Interestingly, the gaps in excision repair processes are small, and would not need a processive enzyme, yet PCNA is required for this repair reaction.^[52–55]^ The exact DNA polymerase that functions in these gap repair reactions is still ambiguous, but gap-filling DNA polymerases can function with PCNA. Thus, it is possible that the PCNA is acting to attract polymerases to sites of damage, and facilities the function of whichever polymerase most fits the repair job at hand.

## CONCLUSIONS AND PROSPECTS

Clearly, there are further discoveries to be made regarding RFC–PCNA and the Rad24-RFC–9–1–1 alternative clamp loader mechanism, and these will be informative studies for the future. For example, we still do not know the exact details of how these AAA+ machines function, and an initial recent deep mutagenesis studies of a clamp loader have identified a conserved network among subunits needed for T4 clamp loader function.^[56]^ Furthermore, the Egl1-RFC and Ctf18-RFC (Chromosome transmission fidelity 18) have been established to function in unloading PCNA from DNA.^[18,19]^ However, PCNA clamp unloading is promiscuous, and can also be performed by Rad24-RFC, RFC, and even just a Rfc2-Rfc5 heterodimer,^[57]^ bringing into question the function of use of a full pentameric RLC for an unloading function alone. Instead, we suggest that RLCs may recognize more specialized different DNA structures for PCNA than the canonical RFC. It was recently shown that Ctf18-RFC binds the leading strand Pol *ε*,^[58]^ and that it may load PCNA for function with Pol *ε* suggesting that Ctf18-RFC may play a more specific role for leading strand synthesis.^[20,21]^ While Ctf18-RFC is not essential,^[59]^ and thus is not likely needed for the replisome, studies of Ctf18 mutants reveal that Ctf18-RFC is important for full activation of the DNA damage checkpoint, and may be important during certain stages of replication, perhaps involving cohesion.^[60–62]^ In overview, it is possible that each clamp loader binds/functions within different DNA metabolic processes to fulfill specific niches in DNA replication and repair programs. Both the mechanism and the physiology behind RFC and RLCs are among the many exciting questions that remain to be addressed in future studies.

## Figures and Tables

**FIGURE 1:**
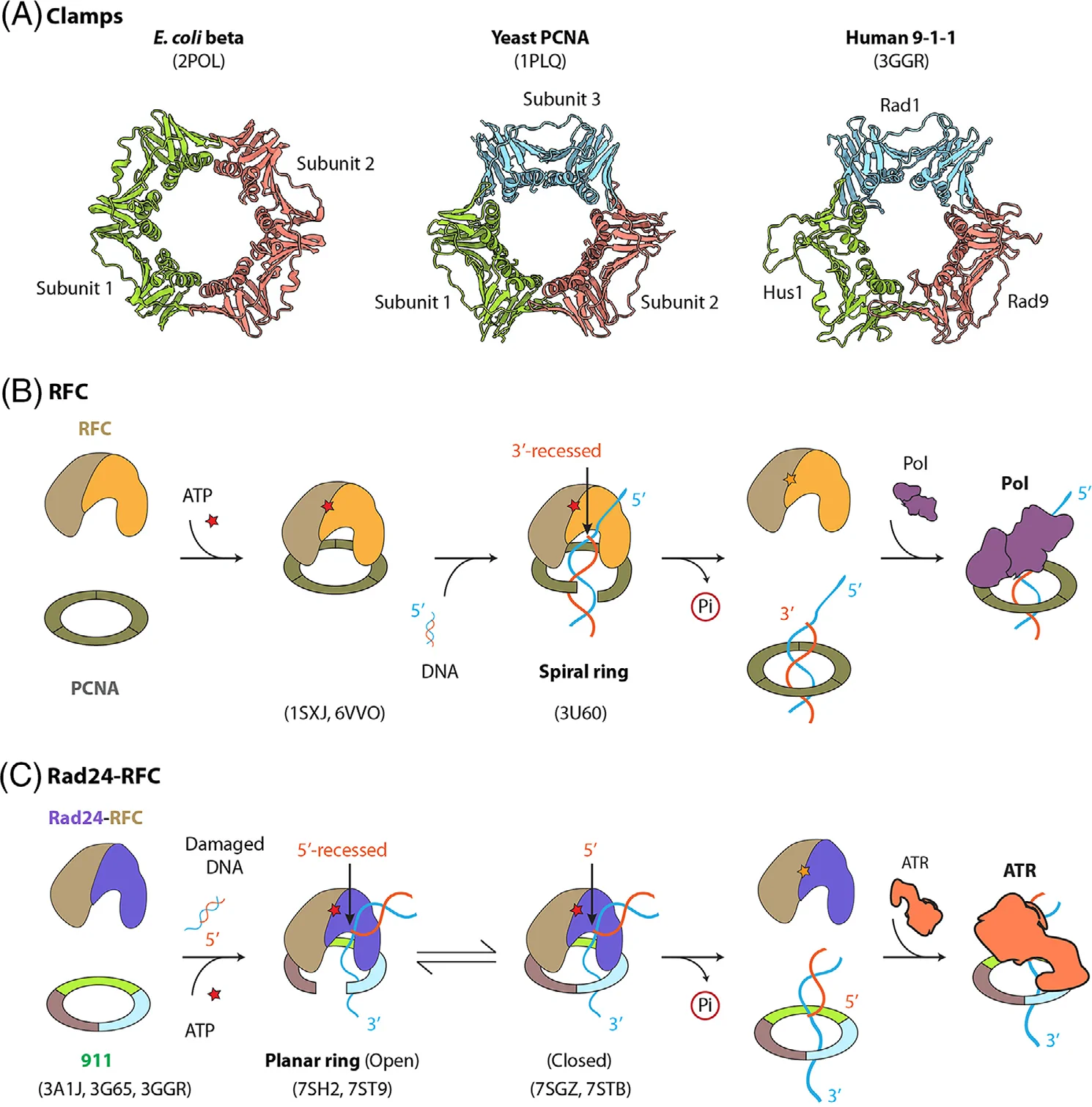
Clamps and illustration of clamp loader function for replication factor C (RFC) and Rad24-RFC. (A) Comparison of clamp structures from a bacterium (*E. coli*), PCNA (eukaryote), and PCNA (archaea). The PDB entries are given in parentheses. (B) Illustration of PCNA clamp loading by RFC for function with a DNA polymerase in contrast to (C), which illustrates 9–1–1 clamp loading by Rad24-RFC and use of 9–1–1 to activate the ATR kinase. Panels B and C are adapted from figure 2 of Zheng et al.^[46]^ See text for details

**FIGURE 2:**
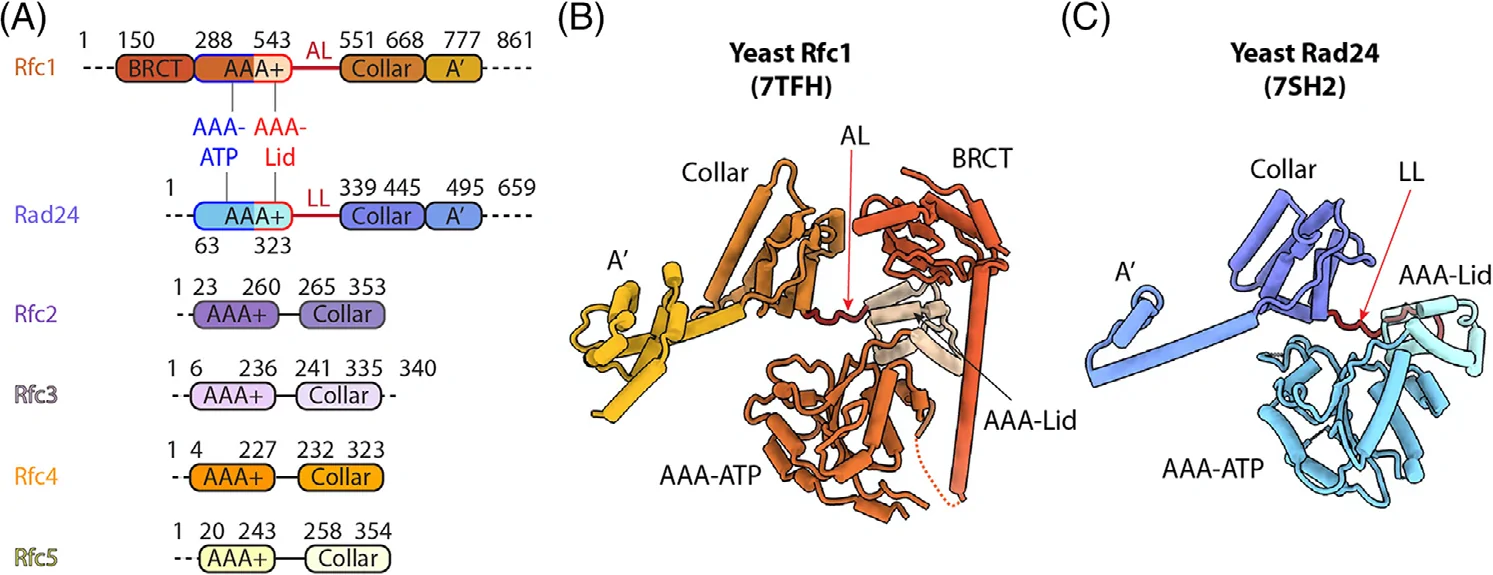
Domain architecture of clamp loader subunits in replication factor C (RFC) and Rad24-RFC. Illustration of the domain architecture of clamp loader subunits. (A) The small subunits, Rfc2–5, are each composed of only two regions, the AAA+ domain having the ATP binding and lid subdomains, and the C-terminal collar domain that tightly holds clamp loader subunits into one pentameric particle. The large “A” subunits of Rfc1 and Rad24 also contain the domains of the small subunits but have additional domains. Rfc1 contains an N-terminal BRCT domain to bind the 5′ end of a recessed DNA gap, and a C-terminal A′ domain that binds the clamp and fills in the “A-gate” between the Rfc5 and Rfc1 subunits. Opening of the A-gate is needed for dsDNA binding and is coupled to PCNA opening (see text for details). Likewise, the Rad24-RFC contains a similar A′ domain that likely fulfills the same function as the A′ domain of Rfc1. However, Rad24 also contains a C-terminal coiled-coil domain known to bind RPA, which may provide it with additional functionality. Adapted from figure 1a of Zheng et al.^[46]^ (b) Domain structure of Rfc1 adapted from figure 2 of Zheng et al.^[32]^ (C) Domain structure of Rfc24 adapted from figure 2 of Zheng et al.^[46]^

**FIGURE 3:**
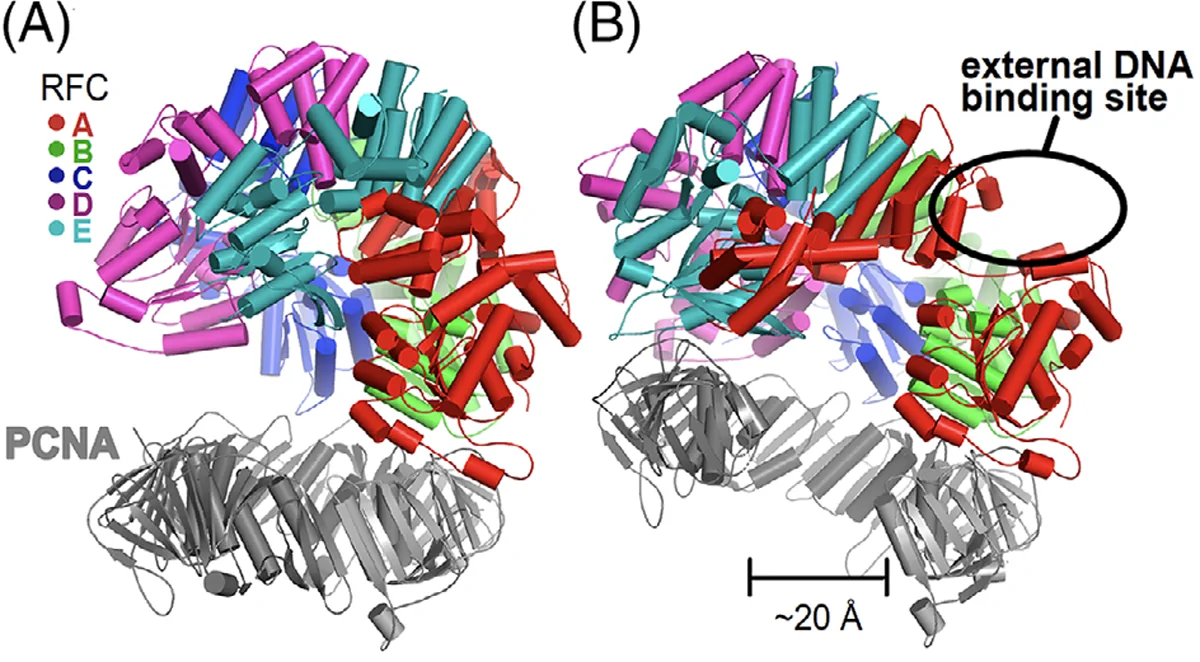
Replication factor C (RFC) and PCNA cooperatively open in concerted “crab-claw” motion. (A) RFC first binds PCNA with both proteins in the closed conformation and the ATPase sites inactive. (B) PCNA opening is accomplished through a large conformational change in RFC that opens the A-gate, exposing the central chamber and a cryptic external DNA binding site. PCNA opens into lockwasher shape with an opening large enough to directly bind duplex DNA

**FIGURE 4:**
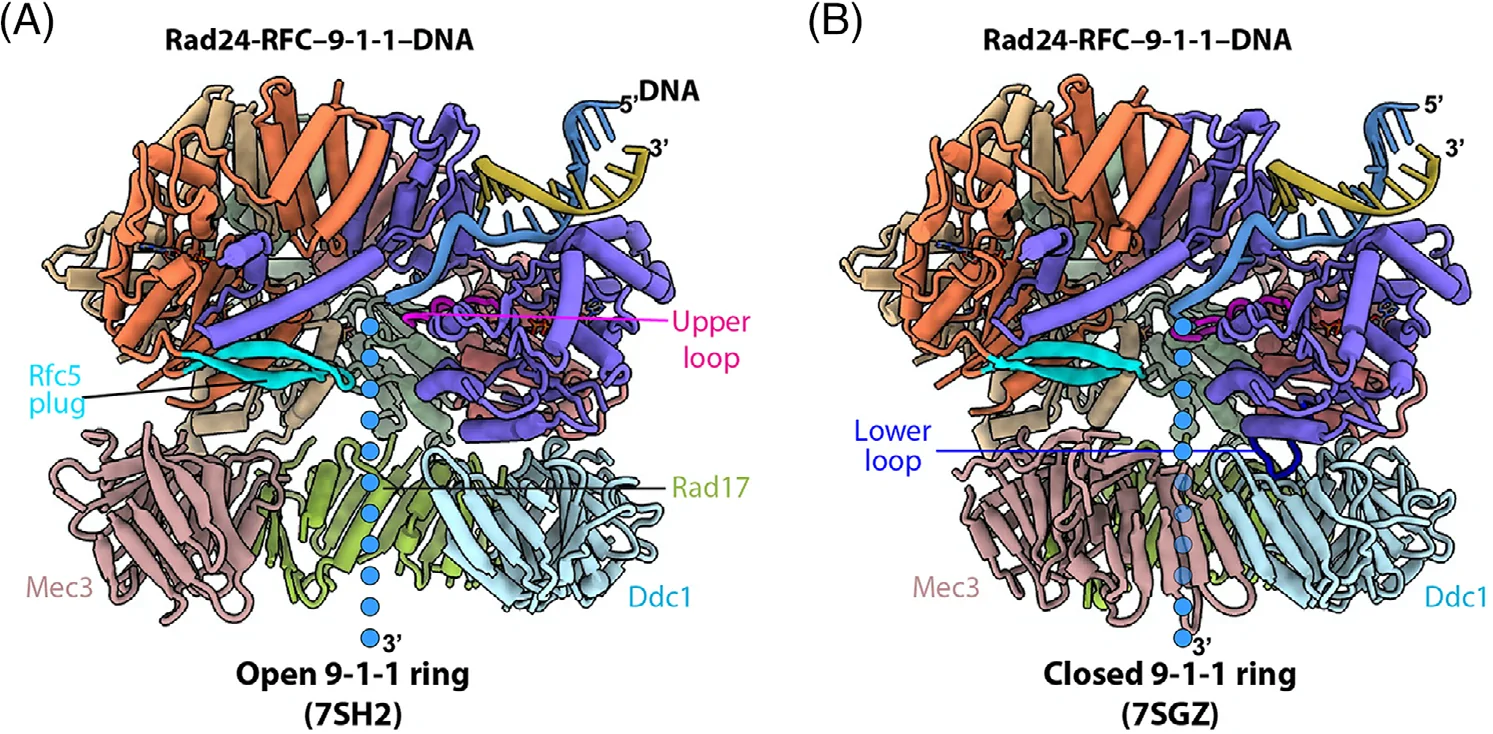
Rad24-replication factor C (RFC) binds a recessed 5′ DNA and threads 3′ ssDNA through the central chamber. This structure of Rad24-RFC with the 9–1–1- clamp further changed the paradigm of clamp loaders in important ways: (1) it opened the clamp wide enough for direct insertion of dsDNA, (2) its primary binding site was outside the central chamber on Rad24 that bound the 5′ end of a DNA, and (3) it placed ssDNA instead of 3′ dsDNA though the central chamber which presumably facilitates ATP firing of clamp loading instead of a need for dsDNA binding. The ssDNA was not visible in this structure, but is presumed to follow the path shown by the blue spheres, as observed in Castaneda et al. (2022). The left panel is the Rad24-RFC–9–1–1_open_–DNA (7SH2), and the right panel is Rad24-RFC–9–1–1_closed_–DNA (7SGZ). The two structures that may restrict dsDNA loading are the Rfc5 plug, and the upper loop (See text for details)

**FIGURE 5:**
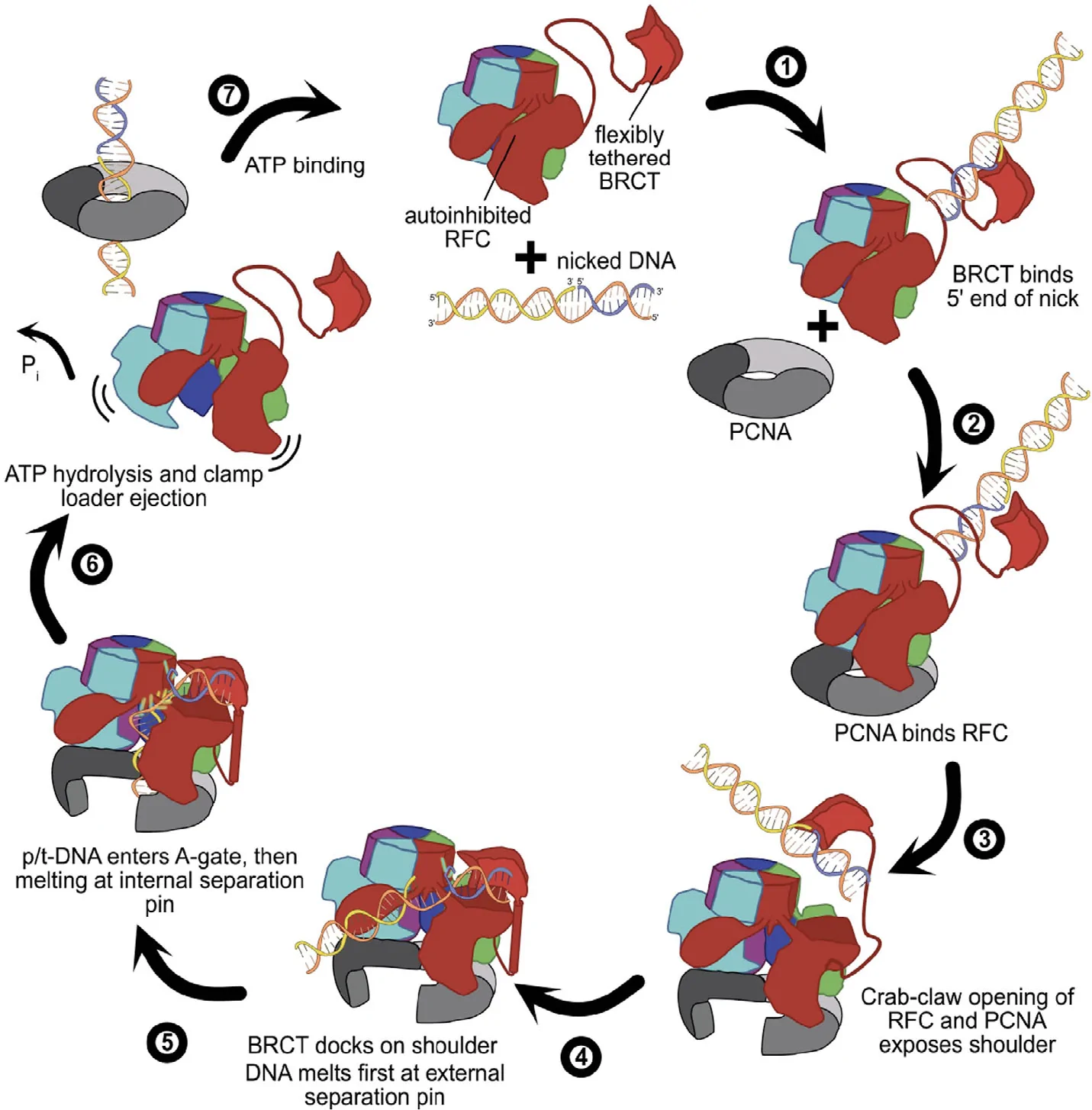
Scheme of replication factor C (RFC) loading PCNA at a DNA gap. (1) RFC–ATP can initially bind to nicked DNA using the flexibly tethered BRCT domain, and then (2) binds PCNA using only three RFC subunits to bind two protomers of PCNA; PCNA is closed. (3) Crab-claw motion of RFC opens PCNA into a lockwasher shape and exposes the external DNA binding site. (4) DNA and BRCT domain dock onto this site, causing partial melting. (5) 3′ primed dsDNA enters the central chamber of RFC, resulting in further melting at the internal separation pin. The open PCNA ring constricts and is in equilibrium with a fully closed PCNA clamp (not shown but see text for details). (6) ATP hydrolysis ejects the clamp loader leaving a closed PCNA on the 3′ side of the gap to attract a polymerase for gap fill-in. Illustration is from figure 6 of Liu et al. (2022).^[50]^

**TABLE 1:** The four distinct clamp loaders in eukaryotes

Clamp loader	A subunit	Common subunits	Other subunits	Clamp	Function
RFC	Rfc1	Rfc2–5		PCNA	Replication, Pol *δ*
Ctf18-RFC	Ctf18	Rfc2–5	Ctf8, Ddc1	PCNA	Replication, Pol *ε*
Egl1-RFC	Egl1	Rfc2–5		PCNA	Unloading PCNA from DNA
Rad24-RFC	Rad24	Rfc2–5		9–1–1	DNA damage checkpoint activator

The nomenclature is the same in human, except for yeast Rad24, which is referred to as RAD17 in human. The unique subunit, or large subunit, is referred to as the A subunit. The smaller subunits, Rfc 2–5, are common among the four clamp loaders. Only the Rad24-RFC is known to utilize a separate clamp at this time (i.e., 9–1–1 heterotrimer). All clamp loaders have five essential clamp loading subunits, but the Ctf18-RFC has two additional subunits that bind other factors, including Pol *ε*. RFC, replication factor C.

## Data Availability

Data from the authors laboratories is available upon request.
